# NSDF: Neuroscience Simulation Data Format

**DOI:** 10.1007/s12021-015-9282-5

**Published:** 2015-11-19

**Authors:** Subhasis Ray, Chaitanya Chintaluri, Upinder S. Bhalla, Daniel K. Wójcik

**Affiliations:** National Center of Biological Sciences, Tata institute of Fundamental Research, Bangalore, India; Nencki Institute of Experimental Biology, Warsaw, Poland; National Institutes of Health, Bethesda, MD USA

**Keywords:** Data sharing, Analysis tools, Visualization, Simulations, Data format

## Abstract

Data interchange is emerging as an essential aspect of modern neuroscience. In the areas of computational neuroscience and systems biology there are multiple model definition formats, which have contributed strongly to the development of an ecosystem of simulation and analysis tools. Here we report the development of the Neuroscience Simulation Data Format (NSDF) which extends this ecosystem to the data generated in simulations. NSDF is designed to store simulator output across scales: from multiscale chemical and electrical signaling models, to detailed single-neuron and network models, to abstract neural nets. It is self-documenting, efficient, modular, and scalable, both in terms of novel data types and in terms of data volume. NSDF is simulator-independent, and can be used by a range of standalone analysis and visualization tools. It may also be used to store variety of experimental data. NSDF is based on the widely used HDF5 (Hierarchical Data Format 5) specification and is open, platform-independent, and portable.

## Introduction

Like in most other fields of science, computational modeling and simulation have become indispensable part of research in neuroscience. The capabilities of computing machinery have steadily increased and the size and complexity of computational models (Kandel et al. 2013) and neuromorphic simulations (Mead 1990; Poon and Zhou 2011; Furber et al. 2014) closely follow the limits of hardware. This growth in scale and complexity also applies to the data generated from such models, and so the storage and management of data from in silico experiments have become common challenges and are recognized as a current challenge in the field (Sejnowski et al. 2014). An ideal format for simulation data would be simple, efficient, flexible, and embody complete information about the originating simulation and simulator. The development of NSDF reflects trade-offs between each of these design goals, closely informed by a broad range of use cases.

Due to their simplicity, plain text formats like csv (comma separated values) are ubiquitous for data storage. Unfortunately they are inefficient in terms of storage space and do not allow any structuring beyond rows and columns as in a spreadsheet. There are many proprietary binary formats associated with specific software, like MATLAB, or hardware, in both basic and clinical neuroscience, which allow more efficient storage (for examples see list of hardware vendors at http://neuroshare.sourceforge.net/links.shtml, each vendor typically using its own proprietary format).

Proprietary formats being closed source in general result in software lock-in and hinder data sharing. One approach to address this issue involves software tools to convert data from one format to another. The Neuroshare API (G-Node 2004) and NEO (Garcia et al. 2014) are attempts in this direction. Several solutions for universal format for storage of experimental data, with different goals and design principles, have been proposed before (EDF (Kemp and Olivan 2003), SignalML (Durka and Ircha 2004), XCEDE (Gadde et al. 2012), BioSignalML (Brooks et al. 2011), NDF (Liang et al. 2010)). Additionally, a recent initiative by the International Neuroinformatics Coordinating Facility (INCF) attempts to develop a standard format for sharing data from electrophysiology experiments (INCF 2014b). Thus storage efficiency issues and data sharing can in principle be addressed using existing neuroscience data formats. However, none of these formats can explicitly store simulation specifics, and their flexibility is limited.

Despite many points of overlap, simulation data has rather different requirements compared to data from wet experiments. In computational research cycle testing a hypothesis is accomplished using simulations. In each cycle, simulations are performed and the data generated are analyzed. Then, simulation parameters are changed, and the simulation is re-run. This is repeated until satisfactory results are obtained. With rare exceptions, such as databases of neuron models (Prinz et al. 2003), data in simulations are generated many times and only the final results with optimal parameters are considered results of the simulation. The data generation in these cases is relatively cheap. However, valid and insightful data generation is an expensive aspect requiring researcher expertise. This is different in experiments where ideally all data over multiple sessions, across many animals, are stored. In addition to researcher expertise in designing the experiment, data collection in experiments is expensive. All experimental data are unique, and cannot be re-generated. In simulations data are generated, whereas in experiments data are collected. Therefore, data are inferred differently post storage. Considering common use cases of simulations and experiments we may observe the following differences:

- Simulated data in our experience have many sources recorded over short biological time scales. In contrast, in experiments, the recordings are at high sampling rates over long periods of time and for fewer (compared to simulations) recording channels. This is a distinct requirement for the simulations as often many thousands of sources take precedence over the recorded time.
- In simulations, many details can be accessed simultaneously, for instance, in detailed compartmental models, membrane potentials, individual ionic currents, ionic concentrations etc., can all be relevant. This is an advantage of simulation studies. The equivalent of this in experiments would be simultaneous recordings from multiple measurement modalities which are usually not accessible to such an extent.
- The data stored are often accessed differently in simulations and in experiments. Based on our insights from developing visualization tools and extracellular potential calculations from simulated data it is often desirable to extract a particular property from all the sources at a time step, perform the necessary operations, and proceed to the next time step. However, this is not a common requirement for the experiments, where signals are averaged over many trials to compensate for the low signal to noise ratios.
- Simulation using many thousands of point neurons is a common use case. Typical simulations are run for tens of seconds. These datasets are inhomogeneous and are most efficiently stored as ragged arrays. Researchers may also store the connectivity matrix of neurons or measure the synaptic weight changes in these synapses over time. Modeling diffusion of molecules in 3D volumes have unique requirements where each vertex index is stored and the vertex coordinate information is also necessary. Such requirements are unique and such data cannot be attached as annotated attributes.
- Experiments often have complex temporal structure. A typical neurobiology experiment can have multiple sessions, each with a different animal. Within a session a sequence of different stimuli can be applied to the animal with multiple trials per stimulus. On the contrary, a simulation experiment is usually a single execution of the program where multiple state variables are recorded in parallel in time.

While similar information may need to be stored in experiments as in simulations, usually requirements are different. For example, experiments with high-density electrode arrays will reach high recording scales, also spike detection algorithms use data from multiple sources simultaneously — for such cases, a more refined strategies may be required (See the “Future of NSDF” section). The design decisions made to accommodate simulation related data may seem unnecessary from an experimental data storage point of view, while the lack of necessary provisions in an experimental data format makes it cumbersome for storing simulation data.

Most notably, model specification and simulator environment details are essential for understanding and analyzing the data context. Therefore, individuals and groups often create custom formats for storing simulation data and then a significant amount of work goes into developing analysis and visualization programs for processing data in these formats. This causes duplication of effort in terms of programming. Furthermore, simulation-specific information is often implicitly built into the data and analysis programs. This makes it hard to share the data or reuse the analysis code. However, data sharing is becoming increasingly important for reproducible research. In recognition of this, many funding agencies mandate data sharing. Similarly, many journals require data sharing as part of the publication process (e.g., Nature (Nature Neuroscience 2007; Moore et al. 2011), PLoS (Piwowar et al. 2007; Bloom et al. 2014), Science (Science Magazine 2014)) and a common format will help in review and verification process. Thus a common open format for simulation data will facilitate data-sharing, clarity of analysis and review, and will support an ecosystem of open and portable tools.

Here we propose a format for storing and sharing data from computer simulations in neuroscience. It is based on Hierarchical Data Format 5 (HDF5, The HDF Group (1997a)), which is widely used for scientific data. HDF5 allows flexible yet efficient storage of numerical data in hierarchical structures with ample provision for metadata. The proposed format is general enough to accommodate the variety of data generated from the wide range of modeling approaches and algorithms used in computational neuroscience. It is designed to be self-documenting so that analysis and visualization tools can operate upon it without additional implicit or explicit knowledge of the data source or content. We also provide an nsdf Python module https://github.com/nsdf which facilitates reading and writing of NSDF files. This module is documented at https://nsdf.readthedocs.org/, and can be readily used by tool developers and modelers for their Python based software.

In the next section we discuss considerations which led to the format and present its specification. We discuss the rationale for keeping different structure variants as parts of the format, as well as connect with the present context of neuroscience data storage. Then, we introduce the nsdf Python module. Next we show examples of data files obtained in real use-case scenarios of neural simulations in different storage variants that NSDF allows. Finally, we show benchmark information for reading and writing these variants and their storage requirements.

## Materials and Methods

### NSDF File Format

#### Design Considerations

In designing NSDF we recognized that data saved from simulations have three main purposes:

1. Analysis — the data must be processed through various algorithms and plotted for inspection by researchers. Since input/output is often the most inefficient step in a computer program, it is important that big data should be stored in a format which can be read efficiently. Moreover, for making inferences based on the relationships of various model components, one should be able to map the data to the model.
2. Visualization — large simulations are usually run in batch mode and often the simulation run time is orders of magnitude slower than real time, making it impractical to visualize the simulation online. A common approach is to visualize the simulation using the recorded data after the simulation is over. This can also be useful in teaching. Tools like NeuronVisio (Mattioni et al. 2012), Geppetto (Open Worm 2014), and Moogli (Goel et al. 2014) can read simulation data in specific formats in combination with the model and “replay” the simulation with graphical visualization in 2D or 3D. Here again the efficiency of reading from storage media is of importance. In addition, since data for a large simulation may not fit the available memory, reading data sequentially, and possibly in chunks, is important. Model metadata are critical in order for visualization tools to understand appropriate ways of representing data, and to specify spatial and functional attributes of the model that form part of the display.
3. Storage and sharing — once data has been analyzed to obtain scientific insight, it needs to be archived for future reference. The same data can be studied by other researchers at a later time with same or different tools. This requires that the data format should be portable, self explanatory to the extent possible, and have sufficient metadata for experienced researchers to interpret it.

Rather than developing an entirely new format, we looked for an existing generic file format which could be used as a basis of more refined structure. A hierarchical file structure with efficient storage of large tables of numbers is suitable for our purpose. XML, which is popular for model specification, is text based and therefore inefficient both in storage space and parsing time when it comes to large and complex data structures. HDF5, on the other hand, was developed particularly for storing scientific data of the kind mentioned above. It is flexible, hierarchical, self-documenting and allows efficient reading, writing and storage via chunking and compression of data in a semi-transparent manner. Moreover, all nodes in HDF5 file can have attributes, which allows storage of metadata along with the data.

In this article we propose a scalable and hierarchical structure for storing the types of data commonly recorded in neuroscience simulations. This structure specifically includes a provision for associating the data with the model components from which it was recorded. The data resulting from neuronal simulations can be categorized into static data, time series, and events. Static data include morphologies, connectivity information, biophysical parameters characterizing the membrane, and any other fields which do not vary over the course of a simulation. Time series data can be sampled at regular intervals or at irregular intervals. Events are data that are discrete in time such as spike times. Below we first describe the top-down structure of the file using an example to motivate the design considerations. We then discuss the details of the key components of an NSDF file: the model specification, the data structures, and finally the mapping between simulation entities and recorded data.

#### File Structure

The design goals of the NSDF file structure were to achieve a modular, extensible, and self-documenting format that took advantage of the capabilities of HDF5, and provide efficiency of reading and writing simulation data. In NSDF we separate the model, data and the mapping between model and data into different groups. Groups are HDF5 containers analogous to directories.

**Fig. 1:**
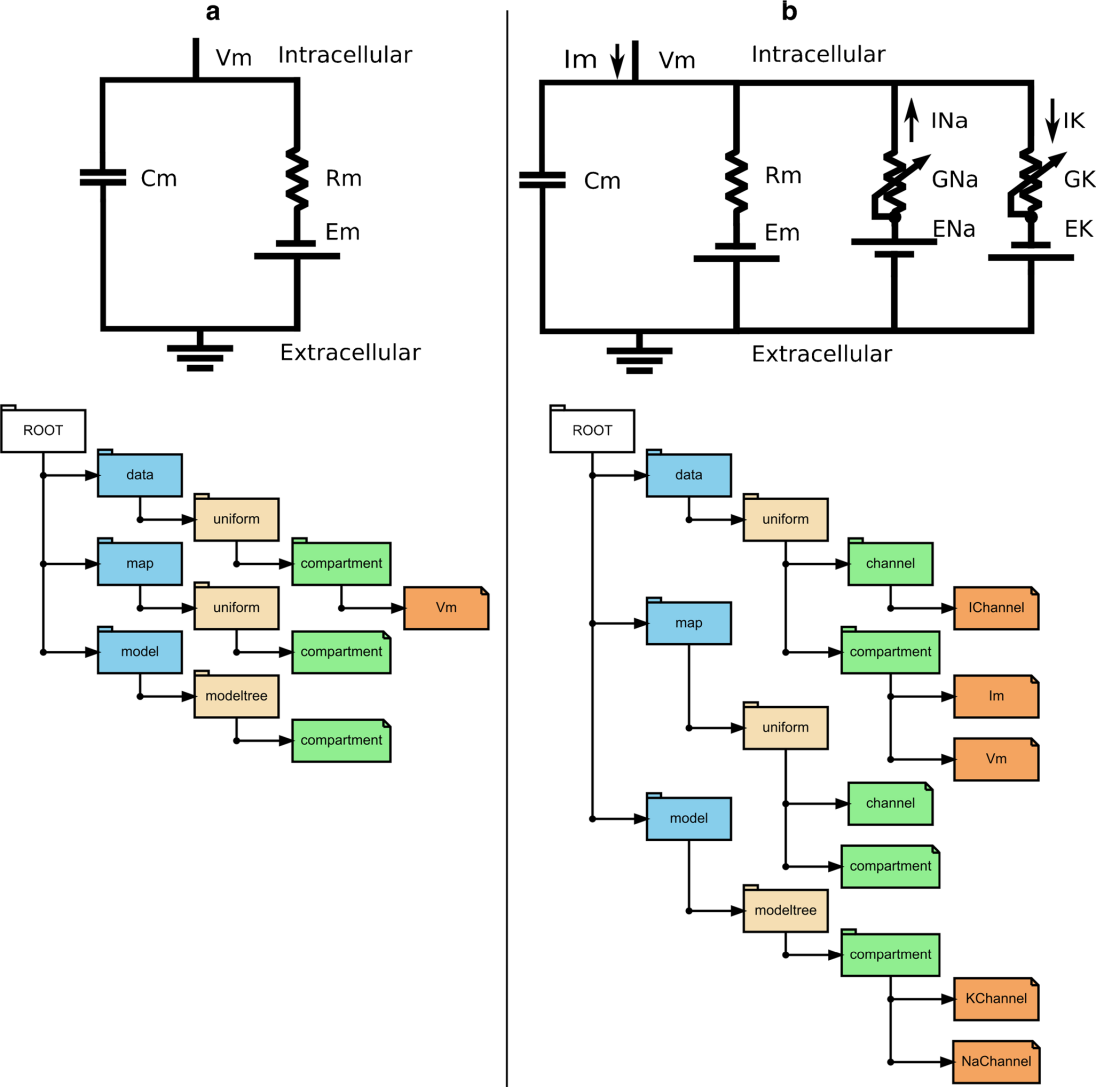
Hierarchy in NSDF file. **a** Shows the hierarchy when saving the data coming from a single compartment Hodgkin-Huxley model. The membrane potential is recorded at uniform time step and is stored under /data/uniform, the corresponding compartment id in map, and the model description under model. **b** The Na and K channels can be considered as two instances of a generic Hodgkin-Huxley channel. We can choose to save the Na and K channel currents as two rows of the dataset IChannel and total membrane current exiting through the compartment as Im and this is shown here

The levels of this hierarchy are as follows:

- Level 0 (Root level): Every HDF5 file has a top level group, called the root group, represented by /. The root group stores a number of recommended attributes about the simulation and system environment used to generate the data.
- Level 1: NSDF has three predefined groups at level 1. These are:
  1. /data, under which all data are located;
  2. /map, storing the mappings between data sources and datasets, as well as sampling times;
  3. /model, a group for storing information about the model from which the data were generated.
  Below we describe the levels under /data. The other groups, /map and /model, are described in later sections.
- Level 2: Data types. There are 4 groups under /data in an NSDF file: static, uniform, nonuniform, and event. These categories were selected based on our experience of different kinds of data from simulations. For example, model parameters and connectivity information are static. Time series values, such as membrane potential in the Hodgkin-Huxley model, may be sampled at uniform (Fig. 1a) or non-uniform intervals. Finally, spiking data are most efficiently stored as events.
- Level 3: Model entities and populations. It is common in simulations to organize the same kind of model components, such as a population of neurons of a given type, into groups. The same fields are often recorded from all members of a population. Such groups of data sources are represented by named groups in level 3 of the NSDF group hierarchy. The importance of this grouping will be explained in the section on mappings.
- Levels 4 and 5: Variables. A simulation may store values for a number of variables from the model components in a group. Each such variable can be represented by a separate node at level 4. The data arrays from individual sources can be clubbed as rows of a 2-dimensional HDF Dataset at level 4 or can be stored as individual 1-dimensional datasets at level 5, in which case the level 4 node is an HDF Group.

As a motivating example, consider storing the output of the Hodgkin-Huxley model (Fig. 1a). In its simplest form, we store the time-series of the membrane potential as the data. Here the model specification only needs to identify the one compartment in the model, and the mapping that joins this compartment to the time-series. At the next level of model detail, we might wish to also monitor channel attributes such as currents. In this case we might either choose to specify the model as an external file, or to provide metadata that specifies the presence of an Na and a K channel entity within the compartment. In the former case, the mapping to the data would take the form of strings that identify the compartment and channels in the external file. In the latter case, the model is represented as a tree (directory-like) structure, with entities for the respective channels, and channel current, conductance, and any other field of interest can be stored as data (Fig. 1b).

**Fig. 2:**
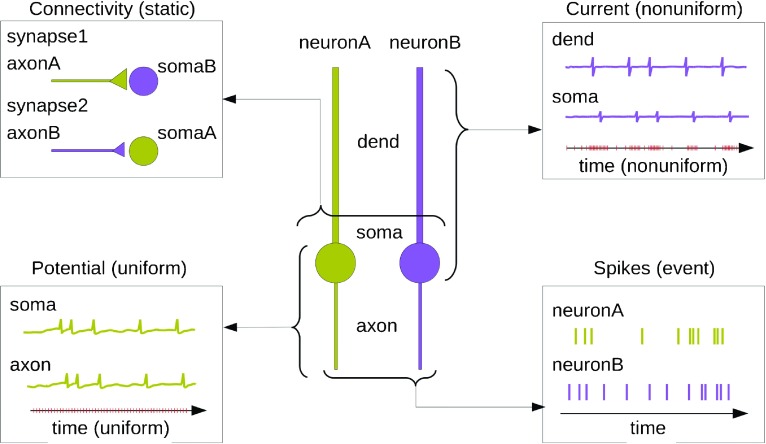
An example of neuroscience simulation data. Consider two neurons, neuronA and neuronB, each comprising three compartments—dend, soma and axon. The neurons are connected by two synapses that do not change over the course of the simulation, and we wish to store this connectivity information (*top left*). Also, the integration time step of neuronA is constant, and we are interested in storing the membrane potential from soma and axon of this neuron (*bottom left*). Further, let us suppose that neuronB is simulated under variable time step, and we are interested in storing the transmembrane currents from dend and soma of this neuron (*top right*). During the course of the simulation, let us suppose that neuronA spikes 11 times and neuronB spikes 13, and we wish to store these spike times (*bottom right*)

We might, at this point, choose to store a richer recording of the simulation output. We could choose to store time-series for Vm as well as currents for each of the channels. These variables could be stored at different time-resolutions. If the simulation were to be solved using a variable-timestep method we should also store the times for each simulation step. Additionally, we can store static data such as the membrane capacitance and resistance, and the channel kinetic parameters. Finally, we could simultaneously choose to store the spike times as yet another time-series. An example elaborating the possible simulation data scenarios is shown in Fig. 2. Each of these kinds of data are supported in NSDF in the data group, as detailed below.

A key feature of this file structure is that it is self-documenting. A human, or a program, reading the contents of an NSDF file, could determine all the entities being recorded, and their relationships to each of the data subsets. Furthermore, through the addition of ontology metadata, specific semantics can be attached to each recorded entity and its associated data.

### Model Specification

The design goals for the model specification section of NSDF were that it be complete and unambiguous in defining which entities were being stored, and that it be flexible in either utilizing external file formats or a default fall-back specification. In either case, the model specification is required only to the extent that it identifies the data. NSDF is not a model definition format, and though it is good practice to include a full model specification, this is not mandatory. NSDF does not support loading and running the model.

NSDF specifies models in two ways. First, NSDF provides a tree-like hierarchical specification of model entities and, at the lowest level, model variables and parameters. Second, models can be specified in a number of distinct non-NSDF formats. In each of these cases NSDF can either store the contents of the model definition files internally as text strings, or refer to external files by URL or filename. The details of these specifications follow.

**Fig. 3:**
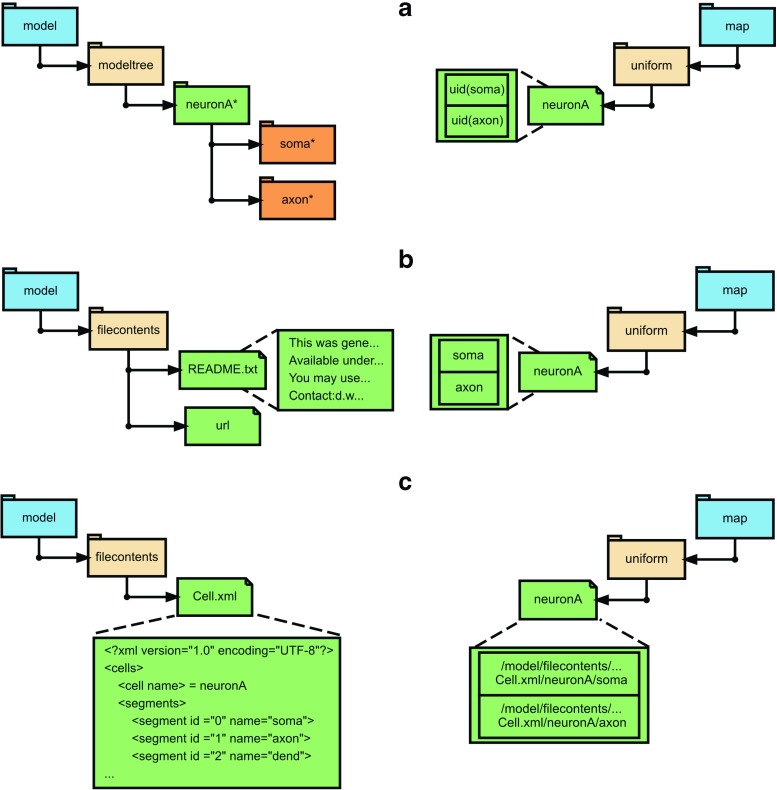
Model descriptions and corresponding map entries in NSDF. Showing some of the possible ways to include model descriptions and the corresponding necessary /map entry in NSDF. The example used here is the same as that in Fig. 2. On the *left* side are entries in /model and on the right side are entries in /map. **a** Case when modeltree is provided for the model like in MOOSE and GENESIS. Here, the tree elements with ’*’ have an uid HDF5 attribute which is used in /map. **b** In this case the model description is not provided within the NSDF file, and the entries in map are given by their unique names. Here there is no link between /model and /map. **c** The XML file that generated the data is provided. In this case, the entries in map correspond to the source path

#### Model Entity Definition Using an Internal Tree Structure

NSDF has a provision for storing model structure internally as a default fall-back option, which is independent of external file formats. The goal of this is to provide assistance to data analysis and visualization applications in a manner independent of model file formats. This is not intended as a full model definition, but rather as a way to uniquely identify each entity whose data are to be stored. In this approach, the group /model/modeltree is used for storing model structure in a hierarchical manner. A tree structure is common in many model description formats including NeuroML, and formats used natively by simulators like MOOSE (Ray and Bhalla 2008) and GENESIS (Bhalla and Bower 1993). This maps well within the hierarchical format of HDF5 files. The hierarchical model structure is stored as a subtree of /model/modeltree with groups representing model components (Fig. 3a). Each group should be named after the model component it represents. Additional attributes are provided for unique identifiers (uid) and ontological terms (ontology). uid can be a URI, XML path or any string that can uniquely identify the data source within the file context. The ontological terms (short form or full URI) can be used for identifying the type of the component based on some predefined ontology like the Computational Neuroscience Ontology (Le Franc et al. 2012) or Gene Ontology Database (Ashburner et al. 2000). Each group in the model tree can be linked to the data recorded from its members through the map attribute, which can be used by software applications to efficiently identify the data collected from this model component or its subcomponents. Apart from these, users are free to store additional metadata in arbitrary attributes of the group.

#### Model Definition Using External Formats

The simplest approach to retain meta-information about a simulation is to store the original model definition file(s). Different simulators use different formats for model description, which may be simulator specific. There are also several simulator independent standard formats like NeuroML (Gleeson et al. 2010), NineML (Gorchetchnikov et al. 2011), NEURON (Carnevale and Hines 2006) and the Systems Biology Markup Language (SBML) (Hucka et al. 2010) for describing cellular, network and biochemical signaling models. NSDF handles external formats in three ways: by storing the original file contents, by providing external file paths, and by linking to model trees in other HDF5 files. The /model/filecontents group is reserved for storing the contents of model description files (Fig. 3b, c). Since complex models are often organized into files distributed in multiple directories, one may replicate the directory structure under this subgroup. HDF5 maps nicely to the UNIX directory structure with groups corresponding to directories and datasets corresponding to files. The text in a file can be stored in the dataset as a string. This approach makes it easy to programmatically replicate the original directory structure used in the simulation along with the file contents. Another way to retain a complete specification of the original model is by storing paths of external files. The /model/filerefs group is reserved for this purpose. This can have one or more string datasets containing paths to model files. HDF5 provides a way to refer to nodes across files via “external links”. The group /model/links can be used for storing external links to include arbitrary subtrees of another HDF5 file storing the model description. This can be a powerful mechanism for linking to large network models with full instantiation stored separately. Thus, these three approaches preserve the entire original model specification along with the recorded data.

Model description is an open problem and all the above approaches have their trade-offs. Storing the contents of the model files is the most complete approach, and is recommended. It is also recommended to store the model tree as this facilitates simulator-independent display and analysis. NSDF supports multiple definitions to introduce flexibility and completeness, but this may also lead to inconsistencies. NSDF specifies a precedence order for interpreting model definitions to avert this. This order is: 1) model tree, 2) file contents, 3) filerefs, 4) filelinks. If necessary, it is also possible to mix different model definitions. Thus a program reading an NSDF file would preferentially utilize the most immediate data specification stored within the file, and then fall back to externally stored model definitions.

In summary, NSDF provides several options for specifying model structure, entities, and metadata, to provide a rich context for the recorded data. This specification allows data analysis and visualization tools to automatically associate data and model.

### Data Structures

The core design goal of the data structure section of the NSDF format is efficiency in file size and speed of reading and writing. We draw upon the hierarchical structure of HDF5 to organize the data into a tree structure, for clear data separation and modularity

#### General Storage Guidelines

All data structures for saving recorded variables in HDF5 files should be stored as double precision numbers. There is no restriction on naming of datasets except those imposed by HDF5. Note that external links to datasets in other HDF5 files can be used in place of datasets. Moreover, HDF5 allows datasets to be stored externally in flat binary files and the NSDF file can act as a wrapper for such data (See Examples). This feature can be useful in parallel environments where a simulation is executed via multiple processes and each process saves its local data in a separate file (Ray and Bhalla 2008).

#### Common Attributes

In HDF5 every node (i.e. group or dataset) can have a set of attributes storing meta-information. In NSDF, a dataset that represents a physical quantity must have a unit attribute associated with it. The unit is stored as a string following SI unit convention with the usual prefixes (ISO 2009) and some additions from SBML (Hucka et al. 2010). Currently SI units are almost universally used in science. However in the area of model specification in neuroscience, there are several proposals for specifying units, each with their merits and problems. For example, NeuroML2/LEMS (Gleeson et al. 2010) specifies a set of units using labels like mA_per_cm2 for mA/cm ^2^ which are associated with dimensional formulas defined in a core set of XML elements using attributes specifying the exponents of the fundamental dimensions. On the other hand, the SBML specification has a set of predefined quantity types with default units, and compound units can be derived from these by specifying the multiplier, scale and exponent attributes. This format, however, is nontrivial for processing by humans or computer programs. NSDF uses an existing unit specification system for saving scientific data. The grammar for this is defined in UDUNITS, the de facto standard units library of netCDF (Unidata Program Center of the University Corporation for Atmospheric Research (UCAR) 2014). This is human-readable as well as compatible with units libraries in several programming languages. For SI symbols with non-ASCII characters, the common convention of writing “ohm” in full. As an example, conductance density can be specified as mS/cm̂2.

In case of compound datasets, an array of strings corresponding to the units of individual columns must be specified and if some of the columns do not represent any physical quantity, such as vertex index number, the corresponding entry in the unit attribute is an empty string.

Another important but optional attribute is ontology. This string attribute stores ontological term for a model component or the variable recorded in a dataset and should be based on some predefined ontology like the Computational Neuroscience Ontology (Le Franc et al. 2012) or Gene Ontology Database (Ashburner et al. 2000).

#### Data Type: Static

This is data related to the specification of the model. It can, for example, include information about the morphology of multicompartmental models, biophysical parameters of the membrane (resistance, capacitance, peak channel conductance, etc.) or in a large network with randomized synaptic strengths one may wish to store the synaptic weights as instantiated at simulation time.

The /data/static group has subgroups representing population level groups (level 3). The organization of a population level group is flexible to the user. Each variable recorded from multiple elements in a population can be wrapped into a 2D dataset where each row corresponds to one element.

For example, to represent morphology information we could store the coordinates of the two ends of every compartment. For an entire cell this would be a 2-dimensional array, with each compartment as a row and the six coordinates as columns. In this case, the row dimension will have the list of source ids attached as the source dimension scale and the column dimension will have the list of labels attached as the field dimension scale stored under /map group as discussed later.

**Fig. 4:**
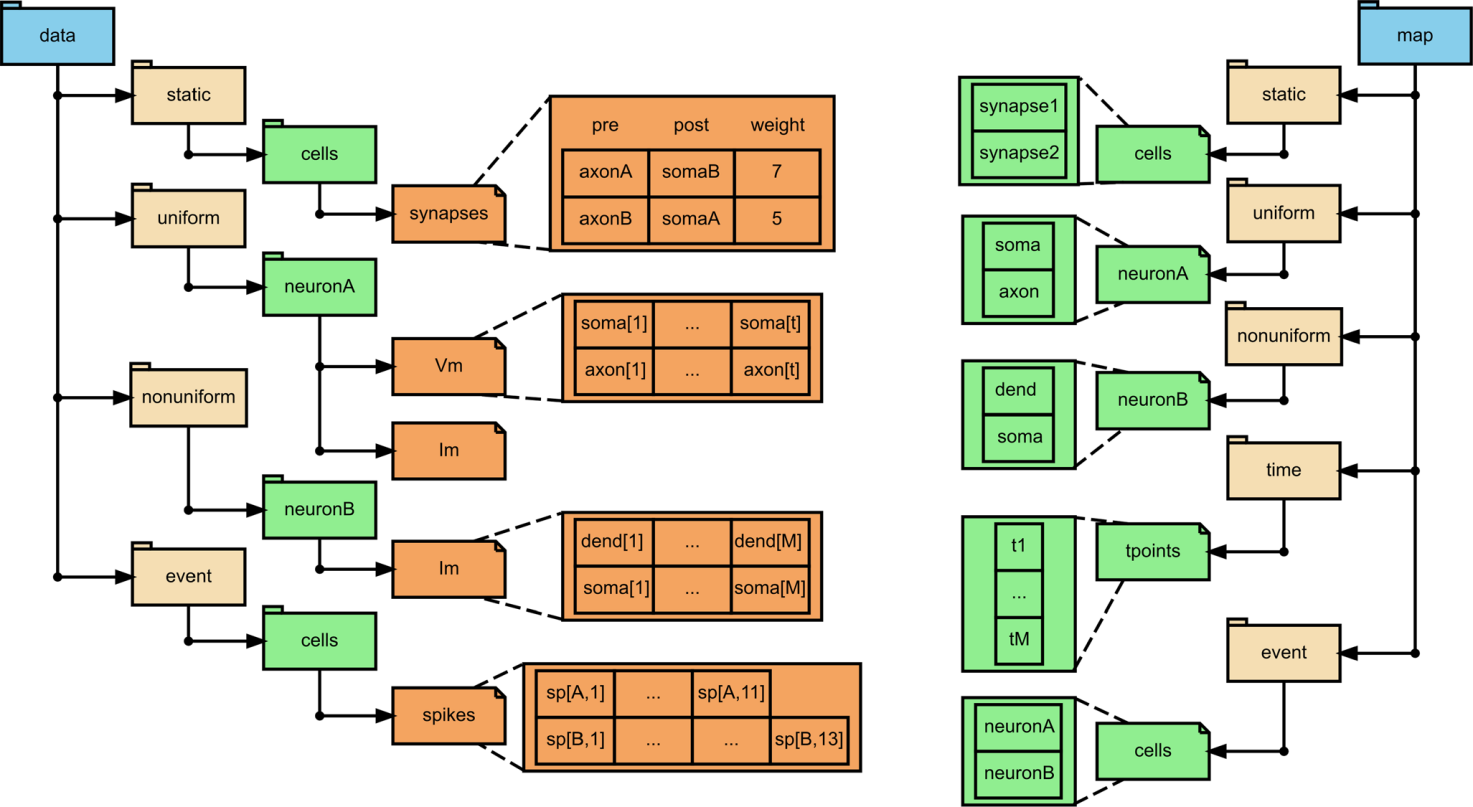
An example NSDF file. This figure illustrates the organization of data in an NSDF file for the example in Fig. 2. Here we assume that the model specification is through an external file, so the local identification of data sources is handled by the map group. The connectivity information, i.e., pre and post synapses and their synaptic weights are stored under /data/static. The corresponding source specification, or uid of the source of this data, is stored under /map/static. The entries in data and map are connected via a dimension-scale which establishes a row-wise mapping between data and map. A similar pairing of data and map is present for time-series data. In this example, the integration time step of neuronA is constant, hence the membrane potential, Vm, and transmembrane current, Im, are stored under /data/uniform. Their corresponding sources are stored under map/uniform. However, since neuronB is simulated under variable time step and the variables are recorded at M nonuniform time points, the transmembrane current Im recorded for this neuron is stored in /data/nonuniform. In this case the sources are stored in /map/nonuniform. Additionally, the time points of measurement are also essential and these are stored in /map/time. The spiking of the neurons constitute discrete time events and must be stored under /data/event. Again, the corresponding sources are stored in the map group, under /map/event. The data shown here can also be stored using alternate NSDF variants. Illustrated here are options corresponding to Table 1, Option 1 (Compound array) for static, Option 1 for uniform, Option 1 (NUREGULAR) for nonuniform and Option 2 (VLEN) for events. Alternate variants for this example corresponding to events are elaborated in the text and illustrated in Fig. 5. The possible model descriptions are illustrated in Fig. 3

Alternatively, it is possible to have compound arrays with named fields as datasets. For example, when storing information regarding synapses one can store the ids of the pre- and post-synaptic compartments, the synaptic strength, time constants, and the synaptic delay in a compound array, with each of the columns named by the corresponding field names. In this case the list of synapse ids correspond to the rows and will be attached as a source dimension scale (see /data/static and /map/static in Fig. 4).

**Table 1:** Variants in NSDF

/data	Option	Variant	Shared Time	Level	DS_0	DS_1	Attributes		
static	1	NA	NA	4	source	^a^	unit	field	
	2	NA	NA	4	source	label	unit	field	
uniform	1	NA	True	4	source	–	unit	field	tstart ^b^
	2	NA	True	4	source	time	unit	field	
nonuniform	1	NUREGULAR	True	4	source	time	unit	field	
	2	ONED	Agnostic	4	–	–	source		
				5	–	time	unit	field	source
	3	VLEN	False	4	source	time	unit	field	
	4	NANPADDED	False	4	source	time	unit	field	
event	1	ONED	NA	4	–	–	source		
				5	–	–	unit	field	source
	2	VLEN	NA	4	source	–	unit	field	
	3	NANPADDED	NA	4	source	–	unit	field	

^a^ compound arrays

^b^ additionally “dt” and “tunit”

For reasons related to efficiency in storage, manipulation, and 3rd party support, NSDF supports more than one way to save simulation data. All the possible options for storing a dataset are listed here. The attributes listed here are mandatory. Additionally, optional attributes such as ontology may also be included. Legend: NA – Not Applicable; DS – Dimension scale; NUREGULAR – a regular 2D array. ONED – One dimensional array variant; NANPADDED – arrays are NaN padded; VLEN – ragged array.

#### Data Type: Uniformly Sampled Continuous Data

This kind of data arises when the variable is sampled (or computed and recorded in a simulation) at constant time intervals. Often the same variable is recorded from many cells in a network (or from many compartments in a cell), at the same time points. The /data/uniform group has subgroups representing population level groups (level 3). Each variable recorded from multiple elements in a population can be wrapped into a 2D dataset where each row corresponds to one element.

For example, if we record membrane potential from neurons *n*_1_,*n*_2_,…,*n*_*P*_, at equally spaced time points *t*_1_,*t*_2_,…*t*_*Q*_, then it is stored in a 2D array, Vm, where Vm[i,j] is the value recorded from neuron *n*_*i*_ at time *t*_*j*_. In this case we can store the start time of simulation and the sampling interval in the dataset as attributes tstart (*t*_1_) and dt (*t*_*i*+1_−*t*_*i*_), respectively. Among several possibilities storing tstart and dt seem to be the simplest: (1) there is no ambiguity about the endpoint, (2) one need not touch these attributes when appending data. The *n*-th data column then corresponds to the sampling time *t*_*n*_:

$$t_{n} = t_{start} + (n-1) * dt. $$

Alternatively, we can store the list of time points (*t*_1_,*t*_2_,…,*t*_*Q*_) explicitly in a dataset in the /map/time group and attach it to the column dimension of Vm as a dimension scale named time. In both cases, the source uids are saved as source dimension scale attached to the row dimension in map/uniform. To avoid ambiguity we recommend to use only one approach in a given dataset, with the more general explicit mapping of times overriding the values of attributes in case both descriptions are present (see /data/uniform and map/uniform in Fig. 4 for a similar membrane potential recording at the compartment level resolution).

#### Data Type: Nonuniformly Sampled Continuous Data

This case comes up in simulations where the underlying numerical method uses an adaptive timestep algorithm such as cvode (Cohen and Hindmarsh 1996). The NEURON (Carnevale and Hines 2006) simulator uses this technique to compute the membrane potential with large time steps when the rate of change is small but with smaller time steps at higher slopes. In such cases it is necessary to explicitly save the time point for each data point. Thus we need two explicit arrays for each time series, one for the data and another for the time points for each data point. However, it is possible that multiple such variables share the same sampling points and thus only one time array will be required for the entire set. In this case, all the compartments of the neuron will be updated at each of the (nonuniform) time steps.

The /data/nonuniform group has subgroups representing populations (level 3). In cases when the same sampling times are shared by all members of the population, the variables recorded from these sources in each population can be collected in regular 2D datasets where each row stores the data from one source and each column corresponds to a sampling time point. We shall refer to this case as the NUREGULAR variant (see /data/nonuniform, /map/nonuniform/ and /map/time in Fig. 4).

In the general case, when the sampling times are different for different sources, the above strategy cannot be applied. NSDF allows three different storage strategies or variants in such cases, as they have different advantages and disadvantages. These variants are referred to as ONED, VLEN, and NANPADDED.

**Fig. 5:**
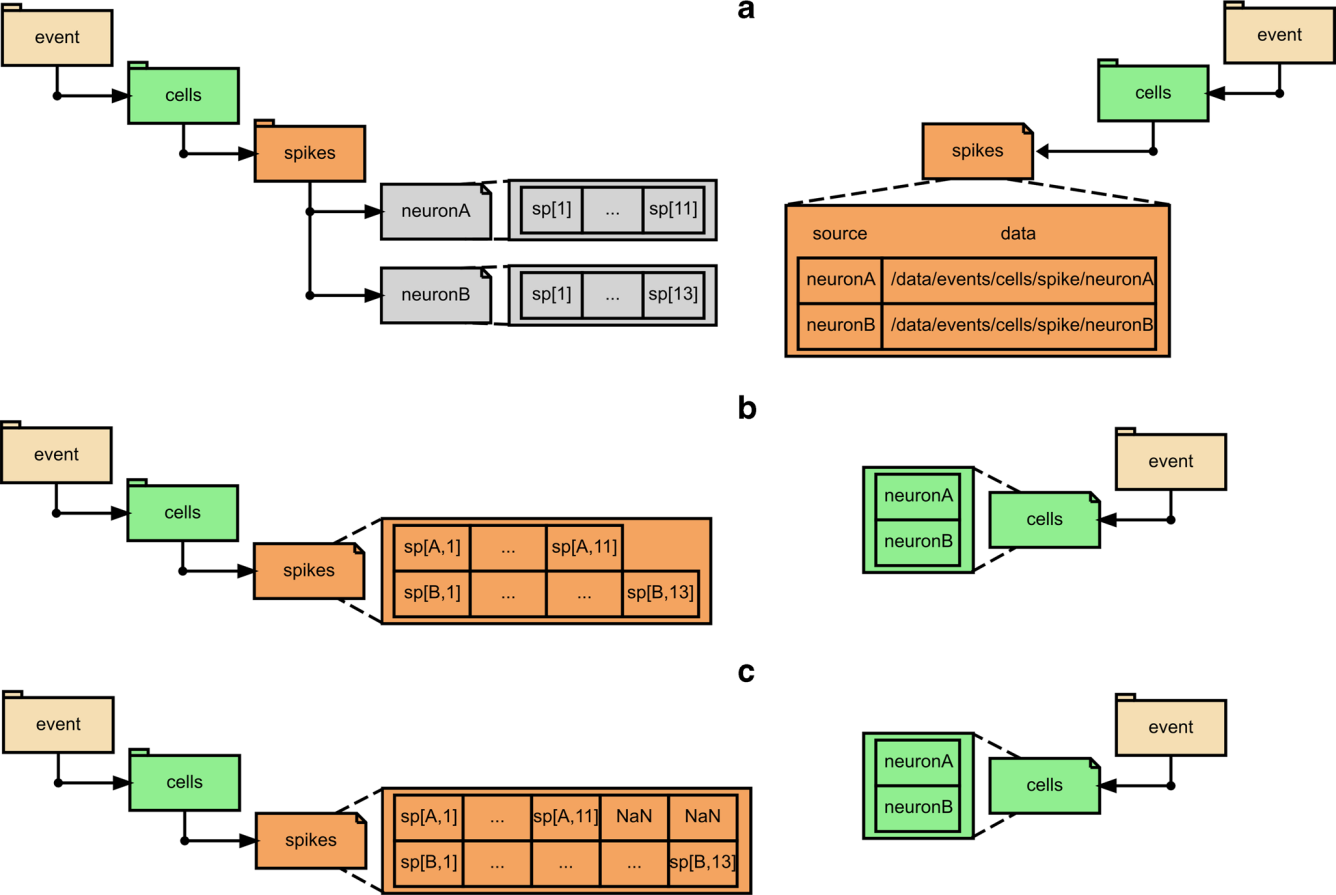
Variants possible in NSDF. Showing all possible variants available for events in addition to the variant shown in Fig. 4 (VLEN). The example used here is the same as that in Fig. 2. On the left side are entries in /data and on the right side are entries in /map. **a** ONED variant, **b** VLEN array (same as in Fig. 4) and **c** NANPADDED array. Additional attributes for the arrays are listed in Table 1. Within a file, all arrays must be in the same consistent variant. These variants are applicable for nonuniform arrays also (not shown). Note that for nonuniform in VLEN and NANPADDED variants, the corresponding source entry in /map/time will also have VLEN and NANPADDED arrays, respectively

In the ONED variant, a single one dimensional dataset is used for each source, and these datasets (level 5) are grouped by variable name (level 4) under the population name (level 3). The mapping between the datasets and the sources is stored under /map in a compound dataset with two fields, source and data, storing the source-uid and the reference to the dataset respectively. Reference to this source-data map is stored as an attribute of the variable group. Each dataset has a dimension scale representing the sampling times associated with it. Moreover, the unique identifier of the data source must be stored in the dataset attribute source. This variant is similar to event data type (see Data type: Event times), with ONED variant (Fig. 5a).

In the VLEN variant, the arrays are left as ragged arrays, i.e., arrays whose rows may not have the same number of columns. HDF5 considers this as a one dimensional dataset each of whose entries is another one dimensional dataset. In this case the list of sources is attached as source dimension scale and the ragged array of the sampling times is attached as the time dimension stored under map. This variant is similar to event data type, with VLEN variant (Fig. 5b).

In the NANPADDED variant, the row lengths are padded with NaN’s to fit into a regular 2D dataset. The row dimension corresponds to the list of sources, which is attached as source dimension scale, and the column dimension has the 2D NaN padded sampling times attached as the time dimension stored under /map. This variant is similar to event data type, with NANPADDED variant (Fig. 5c).

#### Data Type: Event Times

The most common example of event data are spike times. It is possible to maintain an array of size N containing 0’s and 1’s where N is the number of time steps simulated, where a 1-entry indicates a spike event during the corresponding time step. This format is memory-inefficient. The common practice in both electrophysiology experiments and in neuronal simulations is to store only the time of occurrence of each spike as an event time. Since the number of events can be different for different sources over the recording period, we cannot fit data from a population into a regular 2D array. This issue is similar to that addressed by variants ONED, VLEN, NANPADDED for non-uniformly sampled time series. We use the same variants for specifying events (Fig. 5). Note that the NUREGULAR variant is not applicable for events.

Keeping the consistency across different data types, the /data/event group also has subgroups representing population level groups (level 3).

#### Trade-offs

**Fig. 6:**
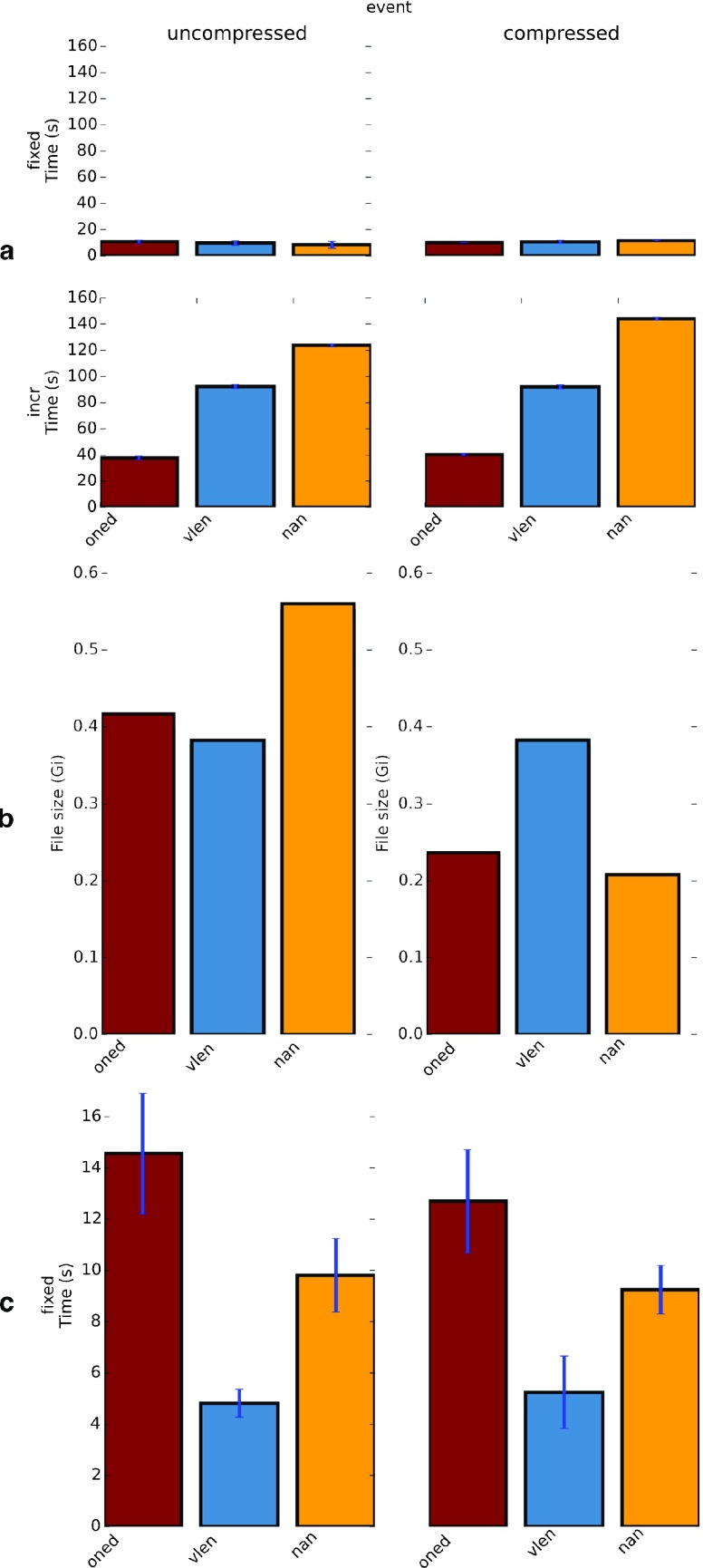
Benchmarks. Shows the benchmarking results for different variants possible within NSDF for events data. **a** Efficiency of writing time, above: one-time writing, below: incremental writing. **b** Efficiency of space, and **c** Efficiency of reading. The benchmarks shown here are for event variants, however, event and nonuniform data have identical storage (ONED, VLEN or NANPADDED) and the plots here capture the general trend. The actual sizes of each entry affect the timings and in our tests nonuniform vlen and nanpadded take almost the same time for incremental writing, which is much more than 1D. These trends do not matter for Uniform case as it is variant free. For details regarding how the data were generated refer to Benchmarks section in the text

We tested all the variants introduced here to store event and nonuniformly sampled data, for efficiency in time (writing speeds — incremental, and one-time write), for efficiency in storage space (uncompressed and compressed), and for efficiency in reading, and present our findings in the Results:Benchmarks section (See also Fig. 6). The results of these benchmarks are uniform over all variants indicating comparable performance. Each of the variants has different limitations and advantages. We believe that there are merits in keeping all these variants.

The advantage of using the ONED variant is that it is the most generic and accommodates future use cases we do not foresee. It is also the most efficient form when writing data incrementally at present. However, it may not make optimal use of HDF5 capabilities.

The advantage of VLEN variant is that it is space efficient even without compression, and reading can be implemented efficiently. However, the issues with this are (1) not every common software package implementing HDF5 supports them, and (2) those that do may not allow appending data in an efficient way at present. As ragged arrays are part of HDF5 specification, it seems likely that this format may be better supported in the future.

The advantage of the NANPADDED variant is that it can be read as a regular 2D array, and it is well supported by other software, (e.g. pandas and pytables). However, heavily NaN padded arrays are large and do not compress well. Further, this format may need extra checks when reading the data to avoid passing NaNs to downstream analysis code. Also, when storing data in an incremental fashion, the algorithm has to search for the end of valid data and start of NaN for each row. This adds to the time-complexity of appending data.

#### Summary

Of the three NSDF components — model structure, data, and mappings — it is not surprising that the data component is the most demanding for speed and space. It is relatively easy to define efficient formats for static data and uniformly sampled time-series. Non-uniform and event data present a multitude of trade-offs, and further the underlying software support is itself evolving. NSDF has therefore opted for flexibility in non-uniform and event data description, and we anticipate that it will take extensive real-world use to narrow this down. A quick reference to this section is presented in Table 1.

### Mappings

Data do not have much meaning without information about their origins. However, we recognize that some users may prefer not to store the entire code that generated the data in NSDF /model. Some would rather host their model description in an online repository and merely provide a link to appropriate version of the model, while others may only include specific parameters for the version that generated the data. For these reasons NSDF only recommends and does not enforce that the model description be provided. To base data references on the numerous possible model description formats would be naive.

In our approach, we make a distinction based on what the data are (entries in /data), to what the labels of the data are (entries in /map) and components that generated the data (entries in /model). This would facilitate traversal either from model to data, via the labels, or just between data and their labels. It also serves as a layer to decouple the data from the model description.

It is a common practice in engineering to introduce an additional mapping layer between two components so that they are decoupled and one can be modified without affecting the other. This is in line with switch boards in a telephone exchange or the TCP/IP protocol. The rationale for using a separate tree for mapping is twofold:

1. When the data are stored in a 2D dataset, each row corresponding to one source, the list of sources is attached as a dimension scale which is just another dataset storing the list of source ids. We use the /map tree for storing the dimension scale dataset. A possible alternative is to store the sources as another column in the same dataset. However, this will create a compound dataset, reduce performance, and complicate processing. Moreover, the former choice allows sharing the same source for multiple data (e.g., current and voltage recorded from the same cells).
2. If one starts with a model component and wants to locate the associated data, the “Mappings” layers can help reduce the search space. In particular, for model described in an internal HDF tree (modeltree), we collect the references to the mapping tables [m1, m2, m3, …] containing source-data mapping for the data collected from components [c1, c2, …] and attach it as an attribute of the common parent. Thus a software tool can search the mapping tables in this list to identify the subset of data to look into and avoid exhaustive search of the /data tree (i.e. go through all datasets under /data and check the associated “source” attribute) to identify the data recorded from a particular component.

NSDF is designed to provide the mapping between recorded data and data sources in an unambiguous way. To achieve this mapping, we utilize the HDF5 facilities directly wherever suitable and define higher level data structures where it goes beyond the scope of HDF5 specification. The /map group in an NSDF file stores the mapping between data and sources as well as sampling times where necessary. It is organized to mirror the structure under /data to simplify look-up of data sources (Fig. 4). This scheme also facilitates manual inspection of the data file. The data contents under map are dimension scales (The HDF Group 2005) which are attached to datasets via attributes and object references. The entries in /map allow shared axis for different entries in data/datatype. ie., the entries /data/uniform/population/parameter1 and /data/uniform/population/parameter2 can have the same unique identifier axis. And in this case, the corresponding unique identifier array for these data will be in /map/uniform/population_ids.

NSDF stores unique identifiers of the data sources in source dimension scales. The exact algorithm of defining the unique identifiers is not within the scope of NSDF but we provide a basic facility for generating such identifiers for hierarchical (tree like) models in the nsdf python module. More importantly, whatever the original model description, the unique identifier must be created in such a fashion that it can be mapped back to the original model component it represents.

In case of tree-structured model description, as in MOOSE and GENESIS, the path of an element can be used as the unique identifier (Fig. 3a). This is to ensure that software for analysis and visualization can unambiguously associate the data with the original model specification. In a generic case, a simulation need not have URI-like references to model components. However, it is valid to assume that the data being stored come from a unique source. We identify these sources by their unique names (Fig. 3b).

In other cases when a network model defined in the NeuroML file *network.xml* is stored as a string under /model/filecontents in the dataset network.xml, then a specific compartment in the cell would be specified as 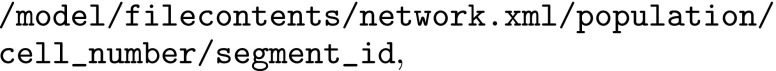 where population, cell_number and segment_id are NeuroML identifiers that uniquely resolve the compartment within the NeuroML file (Fig. 3c).

#### Dimension Scales

DimensionScales (DS) in HDF5 provide a standard way of associating additional information to any dimension of an HDF5 dataset. A DS can label each position along a particular dimension of a dataset. The DS themselves can be datasets containing data of any valid type (e.g., string, float or integer). The connection between a dataset and each of its DS is given by the HDF5 specification (The HDF Group 2005). NSDF utilizes DS to specify the sources and sampling times of recorded data.

In uniformly sampled data in NSDF each row corresponds to a data source. A dimension scale labeled source storing the unique identifiers of the data sources is attached to the first (row) dimension of uniformly sampled datasets. Thus for a dataset entry V[m, n], the identifier of the source will be D[m] where D is the dimension scale attached to the first dimension of V with the label source. For the variables recorded under the group /data/uniform/{population} the DS attached is /map/uniform/{population}. All the datasets under /data/uniform/{population} share the same source dimension scale.

For nonuniformly sampled datasets we provide two different approaches to map data to sources. In the most general case such data are stored in a 1D dataset for each source and the unique identifier of the source object is stored in the source attribute of the dataset. For the reverse mapping an explicit table with the columns source and data is required for each variable recorded from a population of sources where the first stores the unique identifier of the source object and the second contains the path or reference to the corresponding data table. This scheme is illustrated in Fig. 5. On the other hand, if the data are saved in 2D dataset (NUREGULAR, NANPADDED or VLEN variants), then the list of sources can be attached as a dimension scale to the row dimension similar to the case of uniformly sampled data. The same policy applies to event data based on whether they are stored as 1D datasets or 2D datasets.

#### Mapping Sampling Time to Data Points

In addition to mapping the data sources to the datasets and vice versa, one also needs to store sampling times for nonuniformly sampled data. We keep a special group /map/time for storing this information. All sampling times are stored as dimension scales under this group. As per HDF5 Dimension Scale specification, the same dimension scale can be shared by multiple datasets. Thus, even in case of nonuniformly sampled datasets, if they are sampled at the same time points, one can attach a single time dimension scale to all of them. We leave the naming and organization of the time dimension scales up to the users as they can be unambiguously accessed from the original datasets via references.

In case of uniformly sampled data the initial time and the increment stored as data attributes are sufficient to describe the timing of any sample, so one can do without a mapping of sampling times. However, for consistency with nonuniform data, we allow explicit storage of sampling times as a dimension scale as an alternative.

HDF5 dimension scales simplify mapping between uniformly sampled data and their sources, as well as the 2D-dataset formats for nonuniformly sampled and event data. But the 1D storage of the latter two requires an explicit mapping. In this case one has to choose between HDF5 object reference and string path for referring to the dataset. The HDF5 object reference is poorly supported by some of the popular third party libraries whereas the string paths suffer from the risk that renaming the dataset will break the mapping.

The /map group is not only a container for dimension scales, but also a decoupling layer between model and data. Moreover, it can be utilized for efficient traversal from model component to data when the nodes of the modeltree store references to entries in this group. In summary, NSDF specifies a map group that mirrors the structure of the data contents, and utilizes the HDF5 dimension scales construct to define a unique map between model components (data sources) and the stored data.

### Environment Specification

Metadata describing data, like the method of data creation, date and time, and the name of the creator(s), are important for interpretation and identification of the data when sharing it. There are several existing standards for metadata and one can use HDF5 attributes to associate any metadata with any node in an NSDF file. Here we specify some attributes drawing from Dublin Core terms specified in ANSI/NISO Standard Z39.85-2012, augmented with terms applicable to data recorded in an experimental scenario.

Every HDF5 file, and thus NSDF file, has a root group at the top level. This group should have the following attributes relevant to the entire file:

- title : title of the file.
- description : description of the file.
- creator : creator of the file.
- contributor : entities that contributed to the data creation.
- created : time-stamp of creation of file as UTC timestamp in ISO 8601 format.
- license : licensing of the file (optional).
- rights : information about rights over the resource.
- tstart : system time when the simulation/recording was started. This is optional metadata intended to be both human- and machine-readable, this should be date-time string in the ISO 8601 format.
- tend : system time when the simulation/recording was finished. This should be date-time string in the ISO 8601 format.
- software : list of simulation software used for generating the file.
- method : list of numerical methods used (Exponential Euler, Runge-Kutta, etc.).
- nsdf_version : NSDF version number.

The users are free to add further metadata as suitable for specific needs. For example, in simulations using random numbers, it may be useful to store the random number generator algorithm and the seed; in parallelized simulations one may want to store information about the parallel environment. The above is a core set of metadata which should help data sharing and interpretation in the most general case.

### NSDF Library

To facilitate reading and writing data in NSDF file format we developed a Python library which is freely available at https://github.com/nsdf. The documentation of this library is available at https://nsdf.readthedocs.org/. This library is designed to be imported into the Python environment by any Python-compatible simulator. We tested the compatibility of this library on Linux and Microsoft Windows operating systems. Once loaded, all the NSDF file and data handling operations can be transparently accessed by the simulator using the library functions and classes. The NSDF library is structured as a Python module which provides classes for reading and writing data, utility classes for defining the model tree, and several data container classes for organizing data for reading and writing. For large datasets containing numeric values the numpy library (Oliphant 2007) provides efficient data structures and it is the basis of most numeric libraries in Python. Hence we chose this for storing numeric data in memory before they are written to file. There are two popular HDF5 modules in Python: PyTables and h5py, both of which integrate well with numpy. PyTables provides a high level database-like interface to the HDF5 C library and incorporates many optimizations and customizations that are limited to PyTables only. Thus, files written using PyTables may not be portable to other HDF5 readers. On the other hand, h5py is a relatively low level interface to the HDF5 C library. h5py is more complete compared to PyTables as it provides support for variable length datasets, references and dimension scales, which are extensively used in NSDF. For these reasons we used h5py for HDF5 operations. One shortcoming of h5py is that variable length datasets cannot contain 64 bit floating point numbers but hopefully this will soon be addressed by the active developer community.

At the top level NSDF module provides two main classes, NSDFWriter for writing data to and NSDFReader for reading data from an NSDF file. There is a utility class ModelComponent for defining a fallback model tree and several data container classes for organizing data for reading and writing.

The nsdf Python library provides classes for each NSDF data type. Any recorded variable can be temporarily stored in an instance of the appropriate class. Additional attributes appropriate for the type of the variable can also be set: 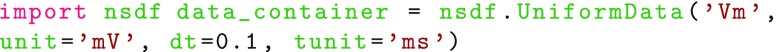


This defines data_container to be a variable for storing ’Vm’ recorded in unit of millivolt sampled at intervals of 0.1 millisecond. NSDF library also provides a basic tree node for defining model structure and it can generate default unique identifier for each model component in the tree: 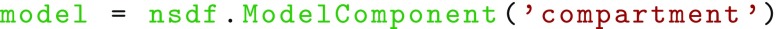


The Vm recorded from this model component can be inserted in the data container: 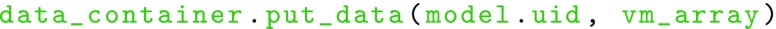


Here vm_array is a numpy array containing data values. Python and numpy have facilities to read data from csv files and data stored in arbitrary HDF5 files can be read using h5py or PyTables modules. The above process can be repeated for all model components that share the same recording parameters. Once all the data sources and their data have been added in this manner, an NSDFWriter object can be created for saving them to an NSDF file. 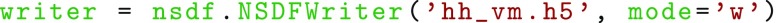


This will create an HDF5 file named ’hh_vm.h5’ in current working directory, overwriting any existing file of the same name. The file level attributes can be specified as a dictionary: 
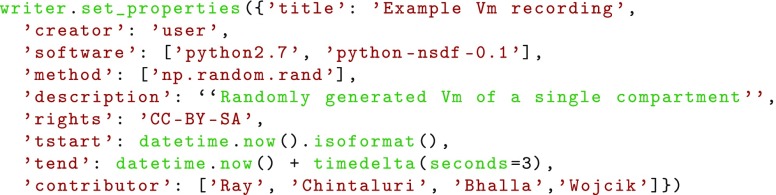


The tree structure of the model can be stored in this file by passing the root node of the model tree: 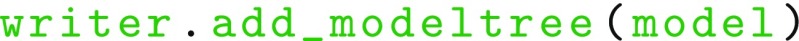


Before adding the data, one must add the sources as a dimension scale: 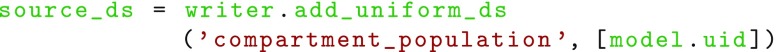


and finally the data container passed to the writer along with the generated dimension scale so that the mappings are made correctly: 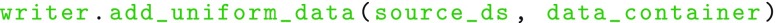


Data writing is by default streaming, which means if add_uniform_data is called again with the same source dimension scale but new data, then the new data are appended to the existing datasets. Similarly, an NSDFReader object can be used for opening an existing file and reading data from it.

At present only reading entire datasets for a variable recorded from a specified population of model components is implemented: 
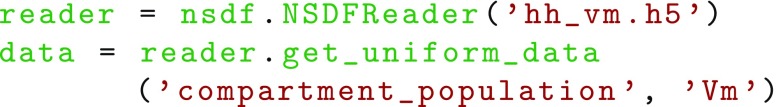


This is the reverse of writer.add_uniform_data and creates a UniformData object containing all the data that was written to the file.

## Results

### Examples

In this section we present several datasets obtained in real case scenarios of neural simulations. When relevant, the files have been prepared in different variants specified above, to compare efficiency of data writing, reading, and storage. The scripts generating some of the files are available as part of the nsdf module in the examples subdirectory and the resulting NSDF files are available at (http://bit.ly/nsdf).

#### *Example 1*

Spike times from a large network

A simulation using Brian simulator (Stimberg et al. 2014) was obtained. This consisted of randomly connected network of integrate and fire neurons (6400 excitatory and 1600 inhibitory) with exponential inhibitory and excitatory currents. The spike times for every cell were recorded for a duration of 10 seconds. This data was inhomogeneous, i.e., the network activity died off quickly, and only few cells continued to fire during the course of the simulation. The different variants possible in NSDF for event type data are shown here.

#### *Example 2*

Membrane potentials from a detailed compartment model

We considered a Layer 5 pyramidal cell (Hay et al. 2011), which models Ca ^2+^ spikes and BAC firing. In this example we illustrate how to save the data obtained from this simulation in an NSDF file. We save the transmembrane currents from all the compartments of the cell, spike times, and the current injections from the patch clamps, artificial EPSPs.

#### *Example 3*

Large detailed compartmental model simulations

In order to see how NSDF model handles large data, we saved data obtained from the largest publicly available single thalamo-cortical column model developed by Traub et al. (2005). This model consists of 3560 neurons of 14 different types. The model was modified to record transmembrane currents from all the compartments, while recording spike times of all the cells. We present samples of data obtained from an example simulation.

#### *Example 4*

Storage of multiscale model simulations

As an example of storing multiscale data, we used a model that incorporates reaction-diffusion as well as electrical signaling in neurons. This model was implemented in MOOSE. The reaction-diffusion model includes Ca ^2+^ and calmodulin (CaM). Calcium influx through ion channels is simulated in the electrical portion of the model, and this is mapped to calcium concentration in the chemical model. In the NSDF file, we store the membrane potential and concentration of Ca ^2+^ from electrical compartments as time-series. We also store the Ca ^2+^ and CaM concentration within all of the reaction-diffusion voxels as they vary over time. We use this example to illustrate the possibility to extend the current uniform 2D datasets to higher dimensional datasets.

#### *Example 5*

Storage of external model description files

As an example of storing external model files with simulated data in NSDF we took a model of a cerebellar granule cell (Maex and De Schutter 1998) which was exported to NeuroML using NeuroConstruct (Gleeson et al. 2007). A script based on MOOSE NeuroML interface (Gilra 2014) loaded and simulated it in MOOSE using its Python interface (Ray and Bhalla 2008). We stored the data and the model using the nsdf library in Python. In this case the entire directory structure of the model, along with the contents of the files are stored under the group /model/filecontent. Since creating uids for model components described in arbitrary model description language is beyond the scope of nsdf, we used MOOSE element paths for uid. However, this has the advantage that the user can easily reproduce the directory structure along with file contents for the model locally and rerun the simulation with appropriate software. This can be very useful for reproducing and verifying results in the data file.

#### *Example 6*

Storing connectivity information

In simulations of a large network of neurons, connectivity information is often crucial. To illustrate saving the connectivity information in NSDF format, we generated a sample dataset, where the presynaptic indices and the postsynaptic indices are stored along with the strength of the synapse. We save this data as a compound arrays under data/static in which the first two columns correspond to the pre and post synaptic indices respectively, and the third is the synaptic weight. This dataset has a source array of synapse index linked as a dimension-scale stored in map/static.

#### *Example 7*

Storing data that is streaming

Long running simulations can generate large amounts of data that cannot be stored in memory. In such cases the simulated data can be streamed to an NSDF file. The exact implementation of when and how the data are flushed is simulator dependent. However, if the nsdf writer finds that the given variable and populations match existing data, then it tries to append the new data to the existing dataset. For demonstration purpose we use the cerebellar granule cell model from example 5 above while appending the data to NSDF file after every 100 steps.

#### *Example 8*

Storing molecular diffusion simulations

To test storing simulations at single molecular level, we used a tutorial from the STEPS solver (Hepburn et al. 2012). This tutorial illustrates diffusion of two molecular species in a cylindrical volume defined by a tetrahedral mesh. One of these molecule types can only diffuse in a restricted part of the volume. In this example, we store the count of each molecular species in every tetrahedron over time. We also store the vertices that constitute the tetrahedron, and the spatial coordinates of each vertex.

### Benchmarks

We carried out extensive benchmarking to characterize the performance of NSDF for different data types, and for different variants for storing the same data. For benchmarking we generated random uniform, nonuniform and event data. For uniform data we generated 100 datasets with 100 rows (sources) and 10000 columns. For each row of nonuniform and event data we chose a random number of columns uniformly distributed between 5000 and 10000. The data were kept identical through all runs by using a fixed seed for the pseudo random number generator. We compared the following cases: uniform data, nonuniform data and event data for each of NANPADDED, VLEN, and ONED variants, with and without compression, each for one-time as well as incremental writing. We used the nsdf library to write the data with cProfile module from Python standard library to deterministically profile the run time of each function and computed the mean and standard deviation of the times from five runs. The results are shown in Fig. 6.

#### Efficiency in Time

Uniform data writing does not depend on the variant, but incremental writing is much slower than one-time writing for the same amount of data. For nonuniform and event data, while all three variants are almost equally efficient for one-time writing, ONED variant is 2–3 times faster than VLEN and NANPADDED variants. Writing the data sources in the map group is more expensive for ONED variant, but this is negligible compared to that of writing the main data and for incremental writing this is a one-time cost at the start (Fig. 6a).

#### Efficiency of Space

In terms of file size VLEN variant produces equal file sizes with and without compression because variable length datasets cannot be compressed in HDF5. NANPADDED variant produces larger files than ONED variant without compression, but with compression it is smaller. Both NANPADDED and ONED variants compress well and produce files that are smaller than VLEN variant (Fig. 6b).

#### Efficiency of Reading

When reading all the data at one go (not streaming), reading is fastest for VLEN, followed by NANPADDED and ONED. One should note that since this is a one-time reading, overhead of traversing through many one-dimensional datasets is included. However in case of incremental reading, a program has to do this traversal only once at the beginning. Also notice that compression shifts the workload from slow I/O system to the CPU, which is much faster, and hence reading compressed data tends to be faster (Fig. 6c).

## Discussion

NSDF is designed to store the most common kinds of neuronal simulation data: model configuration, uniform and non-uniform time-series, and spiking events. The special characteristics of the NSDF format are that it is open, self-documenting and modular. Furthermore, since it is built upon HDF5 it is efficient both in speed and in storage requirements, and supports streaming to disk so that the entire dataset needs not be stored in memory. The basic performance of NSDF relies on the underlying HDF5 library, but our specification also aims to optimize the use of HDF5. For example, by limiting the depth of the data tree we not only keep the data simple for humans to browse using a generic HDF5 viewer, but also make it efficient because traversing a deep tree is more expensive in HDF5 than reading or writing large chunks of flat data. We also utilize the extensibility of HDF5, which is the ability to define new data structures using the existing ones. NSDF itself is extensible in a different sense. First, we emphasize the abstract concept of associating every dataset with the unique identity of its source — which can be any model component or even an electrode if one desires to use it for experimental data. Second, by associating field names in composite datasets and the properties of the source object, we impose a semantic connection, which can be extended to variables with any number of components. Thus the semantics of this extensibility is beyond HDF5 while the low level implementation exploits HDF5 as intended by its designers. The present specification of NSDF is intentionally general and allows multiple possibilities. We expect its use cases to converge on an efficient subset, leading to further refinements in future versions. Refinements can be incorporated in several ways: (1) restricting definitions of datasets, for example, requiring specific names for predefined data, like morphology information under /static/cell/morphology, which will facilitate software that needs to locate such data easily, (2) adding metadata via HDF5 attributes, and (3) object references to entire subtrees in HDF5 storing metadata. In general, NSDF provides the semantics to fit the need for simulation data while trying to utilize the features of HDF5 optimally.

### **Field Context and Ecosystem**

Numerous formats have been used for storage of simulation and experimental data, as outlined in the introduction. The key developments that NSDF delivers are structured metadata and simulator independence. This is enormously important for data sharing and for the emergence of an ecosystem of simulator independent tools. At the time of writing, two NSDF-aware data visualization tools are in development (Moogli (Goel et al. 2014) and dataviz (Ray 2014)) (both in alpha version). Many standard visualization tools, such as the widely used VTK (Schroeder et al. 2003) system, provide a Python interface. Specific visualization tasks could therefore be implemented with small Python wrappers using the nsdf module.

As a way to seed NSDF use, we have two production use-cases which will be made publicly available. In one of our groups, we run simulations of a biologically detailed multicompartment model of single thalamocortical column based on Traub 2005 model (Traub et al. 2005; Gła̧bska et al. 2014). We record all the transmembrane currents from all the compartments under different stimulation protocols. We then compute the local field potentials generated in an in vivo and in vitro context. We use this as ground truth testing data to validate methods of experimental data analysis. These datasets containing transmembrane currents, membrane potentials, spike timing, morphology of the cells in the column and their placement, have been ported to NSDF (release candidate). Analysis scripts for these datasets to compute the extracellular potentials have been developed (alpha version). A manuscript elaborating these datasets is in preparation. In the other group, we run large cortical models also based on the Traub 2005 model, for which we need to store both the connectivity and the spike timing data for further analysis. This dataset too will be ported to NSDF. In addition, we implemented the NSDF writer class in MOOSE (beta version) which can export simulation results as an NSDF file.

Given the Python library interface to NSDF it should be relatively easy to develop further tools for analysis and visualization. Several major neuronal simulators (e.g., BRIAN (Stimberg et al. 2014), NEST (Gewaltig and Diesmann 2007), NEURON (Carnevale and Hines 2006), MOOSE (Ray and Bhalla 2008)) and many data analysis tools provide a Python scripting interface. Again, the availability of the Python library for NSDF facilitates saving and loading data from within these simulators. NSDF also has the potential to interface to analysis and conversion libraries, and other tools for neuroscience data through its Python library, such as NEO (Garcia et al. 2014), NeuroTools (The NeuralEnsemble Initiative 2014), or Geppetto (Open Worm 2014). Each of these tools have their strength in a specific domain and NSDF fills a position complementing them. For example, NEO allows reading and writing data from electrophysiological experiments in different formats using a common object model, thus shielding the user from the idiosyncrasies of different data formats, yet tying her to the Python language and the API of the library. NEO can store its data structures in HDF5 format via the PyTables module, but it is not focused on describing the data and has the limitations of PyTables discussed earlier. However, the NeoHDF5 format is not documented except for the Python API. Also, since the same data can be included in multiple sources (RecordingChannelGroup) and temporal containers (Segments), the generated HDF5 files are not easy for human exploration. NSDF on the other hand provides a language agnostic format which is focused on simulation data, rich with metadata and tries to be as general as possible while remaining specific enough to efficiently address the common needs of simulation data. Data in this format can be shared without conversion and used with different tools capable of handling HDF5. The extensibility of NEO with new input/output functions will allow one to read and write NSDF using NEO and thus provide a way to convert data to/from other formats using the NEO API to the extent possible. A wrapper around NSDF could facilitate the use of standard visualization tools such as VTK (Schroeder et al. 2003). NSDF is based on HDF5, and this already brings in a variety of data visualization tools (e.g. hdfview (The HDF Group 1997b)). NSDF will benefit from further tool development in the HDF5 community, as well as refinements in the HDF5 standards. Data repositories are another emerging aspect of the ecosystem in which NSDF is positioned. The CARMEN project (The CARMEN Project 2006) and INCF dataspace (INCF 2014a) are developed to facilitate sharing of data and shared data analysis, and the availability of a standard for these data will further facilitate exchange.

In the context of data sharing, maintaining provenance of scientific data is a major challenge. Scientific workflow management systems like Kepler (Ludäscher et al. 2006) allow storage of provenance information (Davidson and Freire 2007). There are several projects like NIF (INCF 2015), NIDM (Keator et al. 2013) and Sumatra (Davison et al. 2014) for provenance management in different subdomains of neuroscience. Though it is currently outside the scope of NSDF, as provenance related metadata become standardized, the flexibility of HDF5 will allow easy incorporation of such information into NSDF format.

### **NSDF and Experimental Data**

Could the design principles of NSDF be extended to experimental data? There is an obvious and substantial overlap of NSDF capabilities with those required by experimental neuroscience. Specifically, the major data types (time-series and events) are identical to those for a wide range of electrophysiology experiments. Indeed, while our goal was development of a format for efficient and self-documenting storage of data coming from simulations, the possibility of extending NSDF towards storing experimental data was also factored in its design. The main point of departure is that NSDF data sources are closely tied to data structures characteristic of simulations, whereas experimental data sources are typically instrument-driven. To the extent that experiments and simulations both monitor equivalent biological concepts, the NSDF model specification capabilities could readily be extended to experiments. Thus NSDF shares many design requirements with experimental data formats insofar as the data content is concerned. However, the experimental world is much richer than the NSDF hierarchical vocabulary of biological entities. The specification of experimental data sources is a much more challenging problem, and despite the flexibility of NSDF, the current implementation may not be sufficient for this. In particular, experiments usually have complex temporal structure, e.g. consisting of sessions and trials, data for all of which needs to be organized in a logical way. Technically this can be accommodated either with dimension scales and region references or additional structures in an NSDF file, but is not implemented in NSDF at this point. More importantly, development of adequate metadata for the description of experimental context will require work which should be integrated with existing efforts (NEO, INCF, NWB, CARMEN).

### **Future of NSDF**

While the data structures provided in the present specification of NSDF allow to store spatial and spatiotemporal data, NSDF lacks dedicated data-types for spatial datasets, in particular image sequences and static 3-dimensional reconstructions. These are increasingly important for imaging experiments in different modalities, including but not limited to structural and functional MRI, voltage sensitive dyes, and calcium imaging. Additionally, it is often convenient to treat electrophysiology data from an imaging perspective. For example, data coming from modern high-density multi-electrode arrays with thousands of contacts is often represented as an image where each electrode is a pixel. We anticipate that simulation requirements will eventually also develop to require such datasets as there is a growing interest in simulations of different measurement modalities (Denker et al. 2014). Our use of HDF5 dimension-scales in mapping between data sources and content is entirely consistent with a future extension of NSDF to handle multidimensional image data. Selection of optimal provisions for such datasets will require study of typical uses of such data and benchmarking of different possibilities to develop solution which would facilitate typical imaging analyses.

### **NSDF Governance**

Governance and community engagement are essential for the longevity of any proposed data standard. NSDF will initially be driven by its core developers, and is expected to transition to a community maintainer structure in a couple of years. NSDF is and will remain open-source. The NSDF development approach has been to have a working package and definition for a few well-developed use-cases as a first release. We have already initiated a period of extensive but informal community discussions to strengthen the baseline feature-set developed by the core team. During this period we will engage closely with related efforts especially in the experimental data domain (e.g., Neo, NWB, CARMEN). We will transition to a formal standards development process, as commonly employed by similar communities such as SBML, with a users group meeting and elected maintainers of the standard. We propose to utilize the institutional structures of the International Neuroinformatics Coordination Facility in the long-term governance of the project through this users’ body.

## Information Sharing Statement

The necessary information to store simulation data in NSDF is included in this document. We provide example files in NSDF and these are available for downloading at http://bit.ly/nsdf. We provide a Python based library which is available under GNU General Public License at http://github.com/nsdf/nsdf. The scripts used to generate the above mentioned example files are also provided here. The documentation for this library is available at https://nsdf.readthedocs.org/. This library has been tested on Ubuntu 14.04, and on Windows 7 using Anaconda Scientific Python Distribution.
